# Supplementary material for: Frequent and intense human-bat interactions occur in buildings of rural Kenya
Source: PLoS Negl Trop Dis. 2024 Feb 27;18(2):e0011988. doi: 10.1371/journal.pntd.0011988 (PMC10923417; doi:10.1371/journal.pntd.0011988)
Supplement: S1 Table — Data from these demographics were incorporated into analyses to understand risk factors for direct and indirect interactions between humans and bats in anthropogenic structures. (DOCX) [file pntd.0011988.s001.docx]

Table S1. Demographics of respondents asked about their interactions with bats living in their buildings in rural Kenya. Data from these demographics were incorporated into analyses to understand risk factors for direct and indirect interactions between humans and bats in anthropogenic structures.

| Demographic | Number of people | Mean (Range) |
| --- | --- | --- |
|  |  |  |
| Gender: |  |  |
| Female | 50 |  |
| Male | 52 |  |
| Non-binary/Other | 0 |  |
|  |  |  |
| Age (years) |  | 51.88 (21-92) |
|  |  |  |
| Education Level: |  |  |
| None | 2 |  |
| Primary | 41 |  |
| Secondary | 17 |  |
| Post-secondary | 19 |  |
|  |  |  |
| Property residents (number of people)* |  | 3.89 (0-13) |
| *Two properties contained large unknown numbers of residents and were not incorporated in analysis of resident population size. | | |
