# Supplementary material for: Frequent and intense human-bat interactions occur in buildings of rural Kenya
Source: PLoS Negl Trop Dis. 2024 Feb 27;18(2):e0011988. doi: 10.1371/journal.pntd.0011988 (PMC10923417; doi:10.1371/journal.pntd.0011988)
Supplement: S1 Materials — (DOCX) [file pntd.0011988.s002.docx]

Supplementary Materials 1

Jackson et al. Buildings promote frequent and intense contact between humans and bats in rural Kenya

The survey used to characterize and quantify human-bat interactions in buildings in rural Kenya. This survey was directly translated from English into Swahili and Taita for interviews requiring use of these languages.

1. Who lives on the property?

1a. Respondent demographic data (Age: _____/Prefer not to say, Sex: Male/Female/Other/Prefer not to say, Occupation:_________________/Prefer not to say, Education level:_______________ /Prefer not to say)

1b. Number of people: __________________________________

1c. Number of men (age): _______________________________

1d. Number of women (age): _____________________________

1e. Number of children: _________________________________

1. Structure of the property:

2a. Is there a ceiling in the main building? (Yes/No/I do not know)

2a.i. If yes, how large is the ceiling? (1-3 meters, 3.1-5 meters, 5.1-7 meters long)

2b. How many rooms does the main building? (1-3, 4-6,7+)

2c. How many levels is the house? (1, 2, 3, 4+)

2d. What are the walls of the home made from: (Brick/stone, wood, vinyl, metal, thatch, mud brick, mud plaster, other:__________)

2e. What is the roof of the home made from: (Brick/stone, wood, vinyl, metal, thatch, mud brick, mud plaster, other:__________)

2f. Is the home occupied during the night? (Yes/No/I do not know)

2g. Are there animals kept on the property? (Yes/No/I do not know)

2f.i. If so, what animal species are present and how many? Cow ( ), pig ( ), horse ( ),camel ( ), sheep ( ), goat ( ), chicken ( ), dog ( ), cat ( ), other:__________________

2h. Is there an open water source on the property? (pond, animal trough, other:______________, none)

2i. Are there other structures on the property aside from the main home? (Yes/No/I do not know)

2i.i. Please list: (Barn/livestock building, shed, old unused houses, latrine, kitchen, other:_______________________________________________)

1. Bats on property:

3a. If bats are on your property, where do they roost? Main home, stores, old unused houses, livestock building, toilet, kitchen, trees, other:___________________________

3a.i. If in a building, what part of the building do the bats live in? Roof, ceiling, walls, other:____________________________________________________________

3a.ii. If in a tree, what type (or species) of tree do they live in on the property? Fruit tree (species (if known):_________________) or other:_________________

3.a.iii. Are the bats on the property year-round? (Yes/No/I do not know)

3.a.iii.1 If not year-round, what time of year are bats present? __________

3.a.iv. How many bats are present?______________________________________

3b. How long have you had bats on the property? (1-4 weeks, 1-12 months, 1-2 years, 2-5 years, 5-10 years, 10-20 years, >20 years)

3c. Do all of the bats on the property look the same? (Yes/No/I do not know)

3c.i. If no, how many different types of bats live on the property:_____________

3c.ii. If no, do all of the different bats live in the same area?:_________________

3d. Do you see bats drinking water on the property? (Yes/No/I do not know)

3d.i If yes, where do the bats drink from? ________________________________

3.d.ii If yes, is this the same water source used by livestock or humans? (Yes/No/I do not know)

3e. How many times in the past have you or a household member touched bats on the property? (Every day, every week, every month, every year, never)__________________

3e.i. If not you, has someone in your household touched bats on the property (Yes/No/I do not know) Who:_____________________________________________________________

3f. Have you or a family member received bites from bats on the property? (Yes/No/I do not know)

3f.i. If yes, when have you or a family member ever been bitten by a bat on the property previously? (Within a year, over a year ago, never)

3f.ii. If yes, how many bat bites have you and your family received since you have lived on the property? (1-3, 4-7, 7+) ex:_____________________________

3f.iii. If yes, who on the property has received bat bites previously? _________________________________________________________________

3g. If you or a family member have interacted with bats on the property, what were they doing during these interactions? (flying, roosting/sleeping, biting, crawling, dying, _______________)

3h. How many times in the last year have you or a family member interacted with bat urine/feces on the property: (Every day, every week, every month, every year, never, unknown)

3h.i. If not you, has anyone in your family interacts with bat urine/feces on the property: (Yes/No/I do not know)

3.h.ii. Who else besides you interacts with feces/urine on the property?________________________________________________________

3h.iii. How many times a year do you or a family member clean bat urine/feces from the property: (Every day, every week, every month, every year, never)

3h.iv. If not you, has anyone in your family cleaned bat urine/feces from the property: (Yes/No/I do not know)

3h.v. Who else besides you cleans urine/feces property?____________________

3i. Do you or a family member ever try to remove the bats from the property: (Yes/No/I do not know) Who removes bats from the property? ____________________________

3i.i. If yes, what method(s) do you use to remove bats from the property: (Killing bats, poisoning, scaring bats, blocking access, other:_______________________)

3i.ii. If yes, do the bats return to the property: (Yes/No/I do not know)

3i.iii If yes, why do you choose to remove the bats from the property:

(_________________________________________________________________ _________________________________________________________________)

3i.iv. If no, why do you choose not to remove the bats from the property: (__________________________________________________________________________________________________________________________________)

3j. Do you or anyone in your family find dead/dying bats on the property: (Yes/No/I do not know)

3j.i. If yes, are dead bats removed from the property: (Yes/No/I do not know)

3j.ii. If yes, how are dead bats removed from the property: __________________________________________________________________

3j.iii. If yes, do livestock (i.e., cats, dogs, pigs, etc.) ever contact or consume dead bats found on the property: (Yes/No/I do not know) Which livestock animals? __________________________________________________________________

Table S1. Demographics of respondents asked about their interactions with bats living in their buildings in rural Kenya. Data from these demographics were incorporated into analyses to understand risk factors for direct and indirect interactions between humans and bats in anthropogenic structures.

| Demographic | Number of people | Mean (Range) |
| --- | --- | --- |
|  |  |  |
| Gender: |  |  |
| Female | 50 |  |
| Male | 52 |  |
| Non-binary/Other | 0 |  |
|  |  |  |
| Age (years) |  | 51.88 (21-92) |
|  |  |  |
| Education Level: |  |  |
| None | 2 |  |
| Primary | 41 |  |
| Secondary | 17 |  |
| Post-secondary | 19 |  |
|  |  |  |
| Property residents (number of people)* |  | 3.89 (0-13) |
| *Two properties contained large unknown numbers of residents and were not incorporated in analysis of resident population size. | | |
