# Supplementary material for: Frequent and intense human-bat interactions occur in buildings of rural Kenya
Source: PLoS Negl Trop Dis. 2024 Feb 27;18(2):e0011988. doi: 10.1371/journal.pntd.0011988 (PMC10923417; doi:10.1371/journal.pntd.0011988)
Supplement: Attachment — Submitted filename: Author_Response.docx [file pntd.0011988.s006.docx]

Author Response to Reviewer Comments

Dear Drs. Bowen and Remais,

We are very grateful for the support and thoughtful comments you and the reviewers have provided, which we believe have improved our manuscript. Please find our point-by-point responses below (*italicized text*). Specifically, we have 1) addressed concerns about sample size, sampling method, and population demographics; and 2) broadened the discussion to include content regarding how our results advance our understanding of human-bat interactions, the public health concerns of these interactions, potential measures to mitigate these interactions, and future research efforts in our study area and beyond that could increase our knowledge on human-bat contacts in buildings.

Sincerely,

Reilly Jackson

---------------------------------------------------------------------------------------------------------------------

Three reviewers have evaluated your manuscript and all three indicated that it is well written and presents valuable information and understanding about human-bat interactions. All three reviewers did ask for minor revisions to clarify certain aspects of the manuscript and each provided several suggestions for improvement. Please evaluate these comments, revised the manuscript accordingly and resubmit. Thank you!

*Author Response: We thank the editors and reviewers for their comments on this manuscript.*

Reviewer's Responses to Questions

Key Review Criteria Required for Acceptance?

As you describe the new analyses required for acceptance, please consider the following:

Methods

Reviewer #1: The study sample size of 102 people is small to test the hypothesis given. The authors make reference to the total human population of Taita Taveta county. However, the nature of the county landscape that is under protected forests means that human population are congregated around certain areas. The authors also need to give a better description of the infested buildings for example what were the roofs made of (iron sheets or brick tiles) did the building material play a role in raising the risk for bat inhabiting buildings?

The term rural Kenya is misleading as the county governance structure is devolved meaning the counties have clear urban and rural areas. Was the study conducted in an Urban area like Taita Taveta town? This means that the population also sampled would not be indigenous to the county but people from other parts of Kenya who settle in the town as they are employed or run enterprises and thus require the build rental houses. The study authors need to disaggregate study respondents in two groups indigenous to the county and not indigenous. Literacy level also plays a role in how people approach and tackle issues why was this not a variable on why the respondents chose to live in a build house instead of the traditional roof thatched houses (that may or may have not been also inhabited by bats-did the study come across thatched houses inhabited by bat colonies?)

*Author Response: We thank the reviewer for their insightful comments. We have included language acknowledging our small sample size (Lines 255-265).*

*Regarding building characteristics, we have previously published information on the structural attributes that increase the risk of bat use of buildings (Jackson et al. 2023,* [*https://doi.org/10.1098/rsos.230578*](https://doi.org/10.1098/rsos.230578)*). We have included language pointing readers to this publication for additional details on structural attributes that influence bat presence and that may be altered to help mitigate human-bat contact (Lines 266-276).*

*Lastly, this study was conducted across Taita-Taveta county, much of which is rural. Almost all responses were from outside of urban areas (in rural zones) and all were from indigenous Taita people. We have included language discussing this demographic information (Line 159).*

Reviewer #2: Adequate methods.

*Author Response: We thank the reviewer for their comments.*

Reviewer #3: Objectives were clear enough but the method of identifying the population of individual residences with bat roost/bats living on the property was certainly not clear. It sounds like snowball sampling was the general method, as they describe as 'word of mouth' but the recruitment method needs to be elaborated upon.

Sample size is insufficient, or more description is needed for explaining those who were approached and refused.

*Author Response: We thank the reviewer for their comments. We have included content further detailing the recruitment process (Lines 123-133). Unfortunately, at this stage we cannot increase our sample size. However, we have provided content noting this small sample size and associated limitations (Lines 255-265). We have also included language discussing the occasional refusal of the survey due to fear and cultural associations of bats and witchcraft (Lines 258-262). We do not currently have the number of general refusals to discuss bats given the nature of our sampling method. However, only two respondents started the survey and did not complete the survey, so we feel that we have explained the refusal information adequately.*

--------------------

Results

Reviewer #1: The results are clear and match the analysis plan put forward in the methods section of the paper. However, in Table 1. (Methods used by people to remove bats from buildings). The responses total is 114 yet there were 102 respondents can the authors explain if this was part of a multiple response data analysis?

*Author Response: This question allowed for multiple responses. Therefore, the total number is greater than the number of respondents. We have now clarified this in the manuscript by updating the table caption to “Methods used by people to remove bats from buildings. Attempts to remove bats were common and frequently led to direct contact with bats that could facilitate pathogen exposure. This question allowed for multiple responses from respondents.” (Lines 199-201).*

Reviewer #2: Adequately presented results.

*Author Response: We thank the reviewer for their comments.*

Reviewer #3: Results for this building study are okay, but could be broadened.

*Author Response: We have broadened the discussion of our results following suggestions from reviewers (Lines 211-213, 243-246, 266-279, and 282-286).*

--------------------

Conclusions

-Are the conclusions supported by the data presented?

-Are the limitations of analysis clearly described?

-Do the authors discuss how these data can be helpful to advance our understanding of the topic under study?

-Is public health relevance addressed?

Reviewer #1: The study describes well the zoonotic risk exposure pathways. This is a commendable effort as this was a known fact that had not been documented or quantified. However, the authors missed the opportunity to give a clear policy direction on what type of building design and materials would deter bat infestation. In addition, the failure of authors to describe accurately the unique ecosystem of Taita Taveta means there was a missed opportunity to direct future research to the county as it may be one of the few natural habitats for bats in Kenya.

*Author Response: We have previously answered this question of policy direction in a previous publication (Jackson et al. 2023,* [*https://doi.org/10.1098/rsos.230578*](https://doi.org/10.1098/rsos.230578)*). The focus of the current manuscript is on the nature of interactions between people and bats within these buildings. However, we have added content to the Discussion about mitigation measures, some of which are based on findings from this previous manuscript (Lines 266-279).*

*Regarding the Taita-Taveta ecosystem, we have included descriptions of the system (Lines 114-120) and future directions for research in the county and similar settings (Lines 284-286). However, we would like to note that the bat species encountered in houses are found throughout Kenya (Webala and Patterson 2012) and Taita-Taveta county does not present one of the few natural habitats for bats in the country at this time.*

Reviewer #2: -Are the conclusions supported by the data presented?

Yes

-Are the limitations of analysis clearly described?

Yes, the study discuss the limitations of being survey-based.

-Do the authors discuss how these data can be helpful to advance our understanding of the topic under study?

I think the discussion could develop more on this.

-Is public health relevance addressed?

Yes, but I think this study could discuss further on what can be done in the local context to reduce or mitigate bat-human conflicts, especially after mentioning increase in risk in the foreseeable future.

*Author Response: Thank you for this suggestion. We have now included content discussing what can be done in the local and larger context to reduce/mitigate interactions (Lines 266-279, 284-286).*

Reviewer #3: There is no Conclusion section, just Discussion that ends the paper. Conclusions could be expanded to talk about potential risk mitigation measures on the number of issues raised in Results section.

Authors do not discuss how data can be helpful to advance bat exposure to humans.

and Public health relevance is NOT addressed.

*Author Response: Thank you for this suggestion. We have now incorporated content relating our results to public health concerns (Lines 222-224, 227-229), how our results advance the study of human-bat interactions (Lines 211-215) and possible measures to mitigate human-bat interactions (Lines 266-279).*

--------------------

Editorial and Data Presentation Modifications?

Use this section for editorial suggestions as well as relatively minor modifications of existing data that would enhance clarity. If the only modifications needed are minor and/or editorial, you may wish to recommend “Minor Revision” or “Accept”.

Reviewer #1: Minor edit

Title: Include the county name Taita Taveta and if possible the exact geographical area the study was conducted (sub county and village). The title misrepresents the highly diverse nature of remote locations in Kenya. Taita Taveta has the highest number of protected forests,48, and does not represent the ecology of the arid and semi-arid areas which make up 80% of Kenya's land mass.

*Author response: We appreciate the reviewer’s suggestion to specify our study area. However, this study was conducted across Taita-Taveta county, across three of four sub-counties and across shrublands and some forested areas. Additionally, while this study was conducted in Taita-Taveta, many landscape features and the bat species encountered are found across large parts of Kenya. Therefore, to keep the message broad, we have opted to leave the title as is to appeal to a wider audience. Instead, we have now made it clear early in the abstract that the research setting is Taita-Taveta county (Line 34) and have specified the sub-counties where this work was conducted in the Methods (Lines 114-115).*

Could this be also the reason there are high bat infestation because of the high forest cover?

*Author Response: Previous work in the region (Lunn et al. in revision, 10.22541/au.169029153.39878612/v1) found ~10% of buildings in low elevation areas are occupied by bats in the region, the majority of which were in shrublands. Further, most bats found in buildings here (Jackson et al. 2023) are synanthropic free-tailed bats of the Mops genus, which are aerial, open-air foragers that do not roost in forests. Therefore, the bat use of buildings by these species is unlikely to be linked to high forest cover.*

In the abstract section consider revising the third sentence from the top this will improve clarity.

Bats are associated with several pathogens that can spill over and cause diseases in humans. Rapid urbanization has resulted in the encroachment and loss of the bats' natural habitat, forcing the bats to use anthropogenic structures for roosting.

*Author response: We have addressed this in the manuscript. This section now reads: “Bats are a pervasive synanthropic taxon and are associated with several pathogens that can spillover and cause disease in humans. Urbanization has destroyed natural bat habitat, and many species are increasingly using buildings as roosts.” (Lines 27-30).*

Reviewer #2: L188 - I recommend inverting the sentence, start with `Compared to more common removal reasons,`

*Author Response: Thank you for the suggestion. We have addressed this in the manuscript. This section now reads: “Compared to more common removal reasons, significantly fewer respondents mentioned worries about witchcraft (n = 15), that bats were a general nuisance (n=12) or posed health risks to people (n = 8; χ2 = 123.47, P < 0.01).” (Lines 195-197).*

Figure 1 - Maybe report somehow in this figure the age of respondents for each category or the amount of time respondents have been living at the same place and discuss how those numbers could approximate to a good estimate.

*We appreciate this suggestion from the reviewer. At this time, we do not have information on how long a structure was used by a respondent. However, we have conducted additional statistical tests to evaluate associations between the age of respondents and the reported duration of bat occupancy. Using a multinomial regression, we found that respondent age was not correlated with five categories of bat occupation duration (<1 year, 1-2 years, 2-5 years, 5-10 years, >20 years; p>0.05). Our results show that the age of respondent was negatively correlated with bats occupying structures for 10-20 years, although this relationship was weak (coefficient = -0.08, p=0.045). Given these results, we do not think that age is a good estimate of duration of bat use of a building. Furthermore, the age range was very wide for each category of bat use duration and reports of direct and indirect contact were not associated with participant age (Lines 179-181, 185-187). We have therefore decided not to include this information in the main manuscript to reduce the risk of confusing readers.*

Reviewer #3: THis paper has merit but needs further work. there are topics like the domestic animals' eating of dead bats that should be expanded upon, and this inclusion in 'building'/ediface paper makes me think there could be a follow-up suggested to expand on this narrowly focused questionnaire data than the team is exploiting. Would be a richer paper with a more full-bodied approach and exploration of some of these interface dynamics. Since I see the questionnaire does not include other interface questions, I would heartily suggest further development of results and Discussion section, perhaps suggesting next steps on avenues for exploration, and adding a Conclusions section.

*Author Response: Thank you for your important suggestions. We have now included content expanding the breadth of our Discussion, namely by pointing out how our work advances our knowledge of the field (Lines 211-213), potential mitigation efforts (Lines 266-279), and future research efforts (Lines 284-286).*

--------------------

Summary and General Comments

Use this section to provide overall comments, discuss strengths/weaknesses of the study, novelty, significance, general execution and scholarship. You may also include additional comments for the author, including concerns about dual publication, research ethics, or publication ethics. If requesting major revision, please articulate the new experiments that are needed.

Reviewer #1: The study has notable strengths in documenting and quantifying possible risk pathways for humans sharing living spaces with bats. However the small sample size makes this more an observational study that does not give a high statistical power to make inferences from.

*Author Response:* *We thank the reviewer for their comments on the manuscript. We have included language acknowledging our small sample size (Lines 255-265). We believe that our observations still provide critical information on the importance of buildings as settings for human-bat contact.*

In the discussion section, the authors highlight a study bias of selecting respondents who were unaffected by regional customs about bats. The study failed to describe the study population (indigenous or migrants) so this statement needs to be removed. In addition, if the survey was conducted in an urban area the most likely respondents were not indigenous Taita Taveta community who would be inclined to the cultural fear of talking about bats. The respondents would have been workers from other parts of Kenya who will not be affected by the local cultural belief system.

*Author Response: We have addressed the respondent demographic information by including content stating that all respondents were indigenous Taita (Line 159). Furthermore, we also have worked in this region building trust with this community for several years. We have therefore added content about this relationship in the methods to reduce concerns of bias (Lines 125-128).*

In conclusion, if the authors have more data on the type of housing associated with bat infestations. They can analyse this data and make a policy recommendation on the design and type of roofing that can deter bat roosting especially in an area that is a hot spot for closer bat-human coexistence due the nature of the unique conserved forest landscape of Taita Taveta County.

*Author Response: We have previously published this information in Jackson et al. 2023. (*[*https://doi.org/10.1098/rsos.230578*](https://doi.org/10.1098/rsos.230578)*). However, we have included content in this paper alluding to some of the potential mitigation measures acknowledged in that paper (Lines 266-276). The focus of the current manuscript is the nature of interactions between bats and people within houses.*

Reviewer #2: Dear authors,

Studies like this are much needed so public health authorities can promote guidelines for mitigating bat-human conflict. I believe this draft requires minor changes in order to be considered for publication in PlosNTD.

The suggested changes would be focused on the discussion. For instance, the mentioned stigma could be target of educational projects that could be applied in the region, and some discussion on this topic would be desired. Moreover, I feel like the authors could illustrate more the local context in terms of mentioning existing community projects acting in the region, if there were attempts to provide guidelines for living peacefully with bats. Moreover, the discussion could explore future research demands in the region. Finally, I felt this study could mention how to integrate surveys like this to the One Health approach, by briefly discussing links between human health and biological conservation.

*Author Response: We thank the reviewer for their valuable comments. We have now included content in the discussion following the reviewer’s suggestions, largely focused on addressing stigma through educational projects and community discussions (Lines 270, 276-279), future research needs (Lines 284-286), and integration into a OneHealth approach (Lines 243-246, 284-286).*

Reviewer #3: This paper seems cursory, and a bit superficial, but is of scientific interest, as not much has been done on the bat/human co-habitation interface.

*Author Response: We appreciate the reviewers remarks and have provided additional content to deepen the discussion (Lines 211-214, 243-246, 266-279, 284-286).*

Comments on “Buildings promote frequent and intense contact between humans and bats in rural Kenya” from Dr. Karen Saylors, Reviewer

This is an interesting topic and an important one, but I have concerns about the sample size and lack of description of recruitment methods.

This is a fairly small sample size, if the authors only collected 23 questionnaires in 2021 and 79 in 2022 and 2023 (n = 102): why so small? It would be good to note the number of refusals in the Results section, as the discussion section suggests that there is a negative bias about talking about bats which suggests there may have been significant refusals? This is important to capture in the data in the results section by reporting on total number of surveys attempted, number of refusals, and number of questionnaires completed. What people do NOT want to talk about is also important information.

*Author Response: Thank you, Dr. Saylors, for your comments. Unfortunately, our sampling was constrained by difficulties in finding buildings containing bats: quantitative surveys from this area indicate that only ~10% of buildings are occupied by bats (~8 occupied buildings per km2; Lunn et al. in revision), and even fewer of these are co-occupied by humans. We agree that this sample size is small for testing specific hypotheses on drivers of interactions. The intention of our study was to assess whether people and humans interact in buildings in ways that can promote spillover of pathogens. We believe the data presented here provide clear answers to this largely neglected question that has important public health ramifications. To address these concerns and acknowledge the limitations, we have provided additional discussion regarding our small sample size (Lines 255-265). Lastly, we do not have the exact refusal number due to the nature of how respondents were identified. However, we estimate it to be less than 10 people. Only two respondents did not complete the survey after initially agreeing. Given so few individuals, we believe that our results do capture the trends of human-bat interactions in buildings.*

This paper does not focus exclusively on the buildings that serve to intensify contact between humans and bats, so you might consider adjusting the name.

*Author Response: We have adjusted the name of the manuscript to reflect the results more accurately. The title now reads “Frequent and intense human-bat interactions occur in buildings of rural Kenya”.*

You talk about domestic animals eating bats, but many humans also consume bats. I see this question was not asked in the survey, which is too bad, but you could certainly follow up and expand analysis on the animals eating the bats. Could you follow up with those positive responses to get more information?

*Author Response: In this region of Kenya, bats are not consumed by people (Mickleburgh et al. 2009). Our local partners and collaborators also confirmed this. Instead, bats are largely persecuted here because of their association with witchcraft and the devil, and most people generally want very little to do with them aside from lethally removing them (Webala et al. 2014, Musila et al. 2018). Therefore, we did not ask respondents about human consumption of bats and this was a purposeful decision.*

*That respondents reported several domestic animals consuming bats was an interesting finding from this survey and may be explored in detail as a future research avenue depending on funding availability.*

Normally in the bibliography, you need to list the full citation, rather than say ‘et al.’ This should be redone.

*Author Response: While we agree that this is often the case, the PLOS NTD Submission Guidelines state to use “et al.” after 6 authors.*
