# Supplementary material for: Frequent and intense human-bat interactions occur in buildings of rural Kenya
Source: PLoS Negl Trop Dis. 2024 Feb 27;18(2):e0011988. doi: 10.1371/journal.pntd.0011988 (PMC10923417; doi:10.1371/journal.pntd.0011988)
Supplement: Attachment — Submitted filename: Reviewer comments on Bat human interface paper.docx [file pntd.0011988.s005.docx]

Comments on “Buildings promote frequent and intense contact between humans and bats in rural Kenya” from Dr. Karen Saylors, Reviewer

This is an interesting topic and an important one, but I have concerns about the sample size and lack of description of recruitment methods.

This is a fairly small sample size, if the authors only collected 23 questionnaires in 2021 and 79 in 2022 and 2023 (n = 102): why so small? It would be good to note the number of refusals in the Results section, as the discussion section suggests that there is a negative bias about talking about bats which suggests there may have been significant refusals? This is important to capture in the data in the results section by reporting on total number of surveys attempted, number of refusals, and number of questionnaires completed. What people do NOT want to talk about is also important information.

This paper does not focus exclusively on the buildings that serve to intensify contact between humans and bats, so you might consider adjusting the name.

You talk about domestic animals eating bats, but many humans also consume bats. I see this question was not asked in the survey, which is too bad, but you could certainly follow up and expand analysis on the animals eating the bats. Could you follow up with those positive responses to get more information?

Normally in the bibliography, you need to list the full citation, rather than say ‘et al.’ This should be redone.
